# Sex differences in a murine model of asthma are time and tissue compartment dependent

**DOI:** 10.1371/journal.pone.0271281

**Published:** 2023-10-11

**Authors:** Sergio E. Chiarella, Lyda Cuervo-Pardo, Mackenzie E. Coden, Brian M. Jeong, Ton C. Doan, Andrew R. Connelly, Raul I. Rodriguez, Ashley M. Queener, Sergejs Berdnikovs

**Affiliations:** 1 Division of Allergy and Immunology, Department of Medicine, Northwestern University Feinberg School of Medicine, Chicago, IL, United States of America; 2 Division of Allergic Diseases, Department of Medicine, Mayo Clinic, Rochester, MN, United States of America; 3 University of Florida, Gainesville, FL, United States of America; University of Maryland School of Medicine, UNITED STATES

## Abstract

Asthma is a sexually dimorphic disease with greater documented prevalence and severity in women compared to men. Immunological parameters in mouse models of asthma also exhibit sexual dimorphism, with female mice typically having higher Th2 inflammation than male mice. Despite the standard choice of ovalbumin (OVA) murine models of asthma to study sex differences, there is little consensus across different studies in model design, timelines of sample collection and measured endpoints. To seek agreement in previous reports, we sought to resolve the time course of unfolding sex differences in a standardized 6-challenge OVA murine model of asthma. Immune responses in both lung tissue and airway compartments were quantified by multi-color flow cytometry. OVA-specific immunoglobulin E (IgE) and IgG1 were assayed by ELISA. Several lung tissue markers showed sexual dimorphism as measured by quantitative PCR (qPCR), including cytokines IL-4, IL-5, IL-13 and IL-10 and repair markers nestin and tenascin C. Although females had greater responses in the late phase of the model, male-biased dimorphism was evident in early responses. While females had greater adaptive responses (T and B cells) and higher eosinophils, male data suggested a stronger innate immune response (monocytes, neutrophils). Females had significantly higher OVA-specific IgE prior to the elicitation phase of the model, indicating early sex differences during sensitization. The same parameters frequently had opposing patterns of dimorphism in lung tissue vs. airway.

**Conclusion:**

Sexual dimorphism in lung inflammation is both time and tissue compartment dependent. Spatiotemporal variability in sex differences in a murine model of asthma must be accounted for when planning experiments to model the sex bias in allergic inflammation.

## Introduction

There is a strong age-dependent sex bias in asthma. Before puberty, boys have an increased prevalence and severity of asthma compared to girls [1, 2]. The opposite is true after the onset of puberty, with asthma prevalence being higher in women compared to men [3]. Furthermore, women are more likely to be admitted to the hospital for an asthma exacerbation than men [4–6]. Immunological responses in mouse models of allergic asthma also exhibit strong sexual dimorphism [1, 7], with females typically having higher Th2 cytokine expression and greater airway inflammation. Although ovalbumin (OVA) murine models of asthma have known limitations (intraperitoneal rather than airway sensitization route, dependency on adjuvant, lack of chronicity component of human asthma), these models faithfully reproduce features of adaptive immune response in disease and remain a traditional choice to study the immune mechanisms underlying sex differences in human asthma [8, 9]. Although it is typically reported that females have increased allergic inflammation in these models, endpoints and results vary from study to study. Surprisingly, there is very little consensus across different studies in model design, timelines of sample acquisition, and sampling procedures (**Table 1**).

**Table 1:** Comparison of published sexual dimorphism studies employing ovalbumin models of asthma. The table compares sensitization and challenge protocols used to elicit allergic airway inflammation, as well as the relevant endpoints of each study. i.p.: intraperitoneal; i.n.: intranasal; Ig: immunoglobulin; OVA: ovalbumin; BAL: bronchoalveolar lavage; NS: not significant; ND: not determined. †: authors performed sham-operation or ovariectomy before the sensitization phase.

Study	Mouse strain	Protocol for airway inflammation		Endpoints of interest				
		# of i.p. injections	# of i.n. or nebulized challenges	Eosinophils	B cells	T cells	Type 2 cytokines	Antibodies
Hayashi et al. [10]	BALB/c	2	1	BAL and blood eosinophils (F>M)	BAL lymphocytes (F>M)		IL-4 in splenic cells (F>M)	ND
Okuyama et al. [14]	C57BL/6	2	2	BAL and peribronchial eosinophils (F>M)	BAL lymphocytes (F>M)		BAL IL-4, IL-5, and IL-13 (F>M)	Serum OVA-specific IgE (F>M)
Masuda et al. [11]	C57BL/6	2	2	BAL eosinophils (F>M)	BAL lymphocytes (F>M)		BAL IL-4, IL-5, and IL-13 (F>M)	ND
Riffo-Vasquez et al. [15]	BALB/c	2	3	BAL eosinophils Sham-operated (F>) Ovariectomized (F†)	ND		BAL IL-5 Sham-operated (F>) Ovariectomized (F†)	NS
Melgert et al. [13]	BALB/c	2	5	BAL eosinophils (F>M)	Lung B cells (F>M)	Lung CD4+ T cells (F>M)	Lung IL-4 and IL-13 (F>M)	Serum total IgE and OVA-specific-IgE (F>M)
Carey et al. [9]	C57BL/6	2	5	NS	NS		NS	ND
Warren et al. [17]	BALB/c	1	5	ND	ND		ILC2 IL5 and IL-13 (F>M)	ND
Blacquière et al. [8]	BALB/c	3	8	Lung eosinophils (F>M)	ND	ND	Lung tissue IL-4 (F>M)	OVA-specific IgE (F>M)
Matsubara et al. [12]	BALB/c	0	10	NS	NS		NS	NS
Takeda et al. [16]	BALB/c	2	15	BAL eosinophils (F>M)	BAL lymphocytes (F>M)		BAL IL-13 (F>M)	F>M

Notably, the number of intranasal or nebulized challenges varies substantially between these studies, ranging from one to fifteen [10–19]. There are also significant design differences in studies utilizing different strains of mice. While the more standard three-challenge models are reported for C57BL6 mice, only late phase multi-challenge model sex differences were published for BALB/cJ strain mice. These design differences can lead to seemingly contradictory results. For example, one group reported that females had higher numbers of lung eosinophils compared to males after eight OVA challenges [10], while another group did not find a statistically significant difference after ten OVA challenges [14]. To increase the rigor of sex dimorphism mouse model studies and in an attempt to unify the findings of previous reports, we sought to determine the kinetics of sex differences in a standardized OVA murine model of asthma using two antigen sensitizations and six airway antigen challenges. We decided to use six airway antigen challenges to fully characterize the kinetics of the inflammatory process and phenotypic plasticity of recruited leukocytes. Immune responses in both lung tissue and bronchoalveolar lavage fluid (BALF) compartments were quantified by multi-color flow cytometry, IgE and IgG1 assays and lung gene expression of inflammatory and injury and repair markers. We hope this study will bring to attention the variable and context-dependent nature of sexual dimorphism, and provide a valuable resource for investigators interested in studying sex bias in asthma pathogenesis.

## Materials and methods

### Animals

For all experiments, we used 10 to 12 week old male and female BALB/cJ littermate mice (Jackson Laboratories, Bar Harbor, ME, USA). The Institutional Animal Care and Use Committee (IACUC) of Northwestern University approved all animal procedures (IACUC Study #IS00001710).

### Ovalbumin murine model of asthma

**Fig 1:**
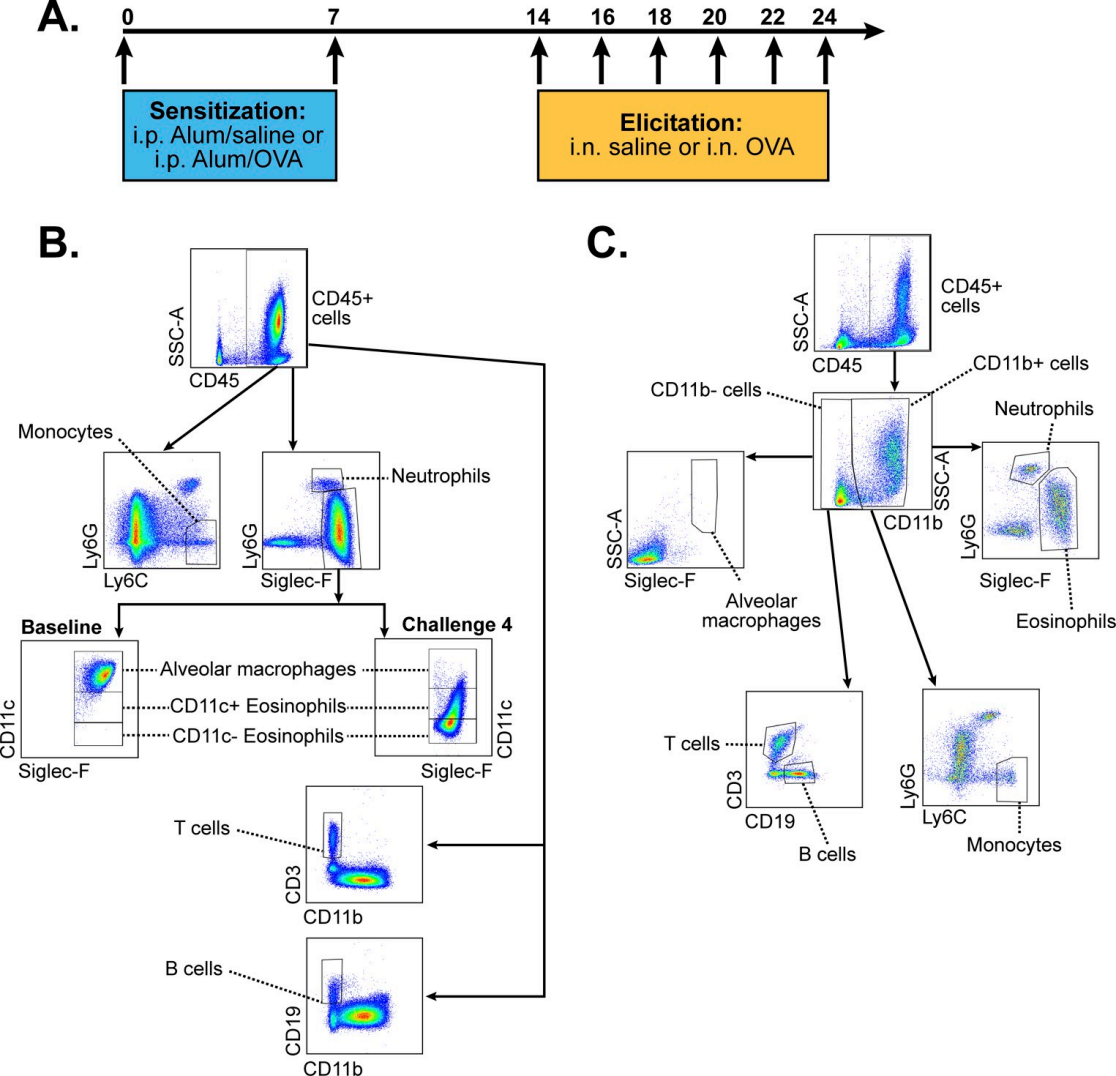
Timeline of treatments and flow cytometry gating strategies. (A) Timeline of intraperitoneal OVA/alum injections and OVA intranasal challenges. (B) Flow cytometry gating strategy to identify and quantify leukocyte populations in bronchoalveolar lavage fluid. (C) Flow cytometry gating strategy to identify and quantify leukocyte populations in whole lung homogenates.

We sensitized mice using two intraperitoneal injections of chicken egg ovalbumin (OVA) grade V (Sigma) (50 μg in alum) on days 0 and 7. Starting on day 14, we challenged mice every other day with intranasal OVA grade VI (Sigma) (50 μg in saline). Different groups of male and female mice received one, two, three, four, five, or six intranasal challenges. Timeline of OVA kinetic model is illustrated in **Fig 1**. The animals were euthanized by carbon dioxide inhalation followed by cervical dislocation. We obtained BALF, lung tissue, and serum 24 hours after each intranasal challenge. We used 4 mice per sex for the baseline timepoint and challenge 6. For all the other timepoints we used 8 mice per sex.

### Bronchoalveolar lavage, lung digestion, and multi-color flow cytometry

We obtained BALF by lavaging the lungs with 1 mL of ice-cold 1X phosphate buffered saline (PBS) through the cannulated trachea. Subsequently, we removed the lungs and digested them as previously described [20]. We stained the preparations for live/dead exclusion with Aqua dye (Molecular Probes) followed by incubation CD16/CD32 Fc Block (BD Pharmingen, San Jose, CA, USA). We added the antibody cocktail directly to the blocked samples and incubated them for 30 minutes at 4°C. We used a BD LSRII flow cytometer (BD Biosciences) to acquire the samples. For multi-color flow cytometry, we used the following flurochrome-conjugated antibody cocktail: (1) FITC-conjugated CD45 (clone 30-F11, Biolegend); (2) APC-Cy7-conjugated CD11b (clone M1/70, BD); (3) PE/Cy7-conjugated CD11c (clone N418, Biolegend); (4) Alexa Fluor 647-conjugated Siglec-F (clone E50-2440, BD); (5) PE-conjugated CD64 (clone X54-5/7.1.1, BD); (6) Alexa Fluor 594-conjugated CD3 (clone 17A2, Biolegend); (7) PerCP-Cy5.5-conjugated CD19 (clone eBio1D3, eBioscience); (8) eFluor450-conjugated Ly-6C (clone HK1.4, eBioscience); (9) Alexa Fluor 700-conjugated Ly-6G (clone 1A8, Biolegend); (10) Zombie Aqua Live/Dead dye (Biolegend). Fluorescent Minus One (FMO) controls were used to define gate boundaries. Compensation and flow cytometry data analysis were performed in FlowJo v10 (FlowJo, LLC). The gating strategies for the identification of BALF and lung tissue leukocyte populations are illustrated in **Fig 1**.

### Quantitative PCR (qPCR)

We isolated mRNA from lung tissue samples lysed in buffer RLT (RNeasy Lysis Buffer for tissues, Qiagen) using RNeasy kits (QIAGEN). 500 ng of total RNA was used in a cDNA synthesis reaction, using a cDNA Synthesis Kit (Quanta BioSciences). The qPCR reactions utilized probe-based qPCR Master Mix (Integrated DNA Technologies [IDT]) and commercially pre-designed and validated primer/probe gene expression assays (available from IDT) for the following genes: cytokines (*Interleukin [IL]-4*, *IL-5*, *IL-10*, *IL-13* and *IL-33)*, chemokines (*C-C Motif Chemokine Ligand [Ccl] 2*, *Ccl5*, *Cl11*, and *Ccl24*), growth factors (*Transforming growth factor beta 1 [Tgfb1]*, and *Insulin-like growth factor 1 [Igf1]*) and tissue remodeling markers (*Caspase 1 [Casp1]*, *NLR Family Pyrin Domain Containing 3 [Nlrp3]*, *IL-1β*, *Fibroblast growth factor receptor [Fgfr] 1*, *Fgfr2*, *Aldolase A [Aldoa]*, *SRY-box 2 [Sox2]*, *Nestin [Nes]*, *Tenascin C [Tnc]*, *Vimentin [Vim]*, and *Periostin* [*Postn]*). The qPCR reactions were run using a StepOnePlus Real-Time PCR System (Applied Biosystems). Gene expression was calculated relative to the expression of GAPDH (housekeeping gene) and was reported as true copies of the gene of interest per 10^4^ copies of GAPDH as previously described [21].

### Measurements of serum ovalbumin-specific IgE and IgG1

We measured serum OVA-specific immunoglobulin E (IgE) and immunoglobulin G1 (IgG1) using commercially available ELISA kits (Cayman Chemical). Lower limit of quantitation was 3.12 ng/ml for the IgE assay and 1.56 ng/ml for the IgG1 assay. ELISAs were performed according to the manufacturer’s protocols.

### Statistical analysis

Statistical significance of all data was determined by two-way (sex, time) ANOVA followed by post-hoc pairwise testing. As a significance level we used an alpha level = 0.05. In all experiments, we tested N = 4 mice of each sex per time point for “Baseline” and “Challenge 6” groups, N = 6–8 mice/sex per time point for “Challenge 1–5” groups”. For example, we had 4 male and 4 female mice in baseline group, and 8 male and 8 female mice for Challenge 5 time point collection in the model, et cetera. Exact sample numbers are detailed in each figure legend. On average, the observed effect sizes ranged from 0.9 (for example, measurements of antibody productions by ELISA, representing increase in antibodies from 0.000375533 ug/ml at baseline to peak of 2176.89 ug/ml after challenge 5) to 0.5 (cell number estimates for flow cytometry results, corresponding to approximately two-fold changes in cell counts in at least one time point relative to baseline). Based on the observed effect sizes, power analysis demonstrated that in most cases, 4–8 samples per group were sufficient to achieve statistical power of 0.8 for an alpha level = 0.05 in resolving these differences. When comparing challenge time point responses (1–6) relative to baseline (for either male or female data) by two-way ANOVA: *p < 0.05; **p < 0.01; #p < 0.001; †p < 0.0001. Gray shaded boxes represent a statistically significant difference (p < 0.05) between males and females at that particular time-point. All data are represented as mean ± S.E.M. (standard error of the mean). Statistical analysis was performed using GraphPad Prism 7 (GraphPad Software, Inc).

## Results

### Sexual dimorphism in lung and BALF leukocyte populations

**Fig 2:**
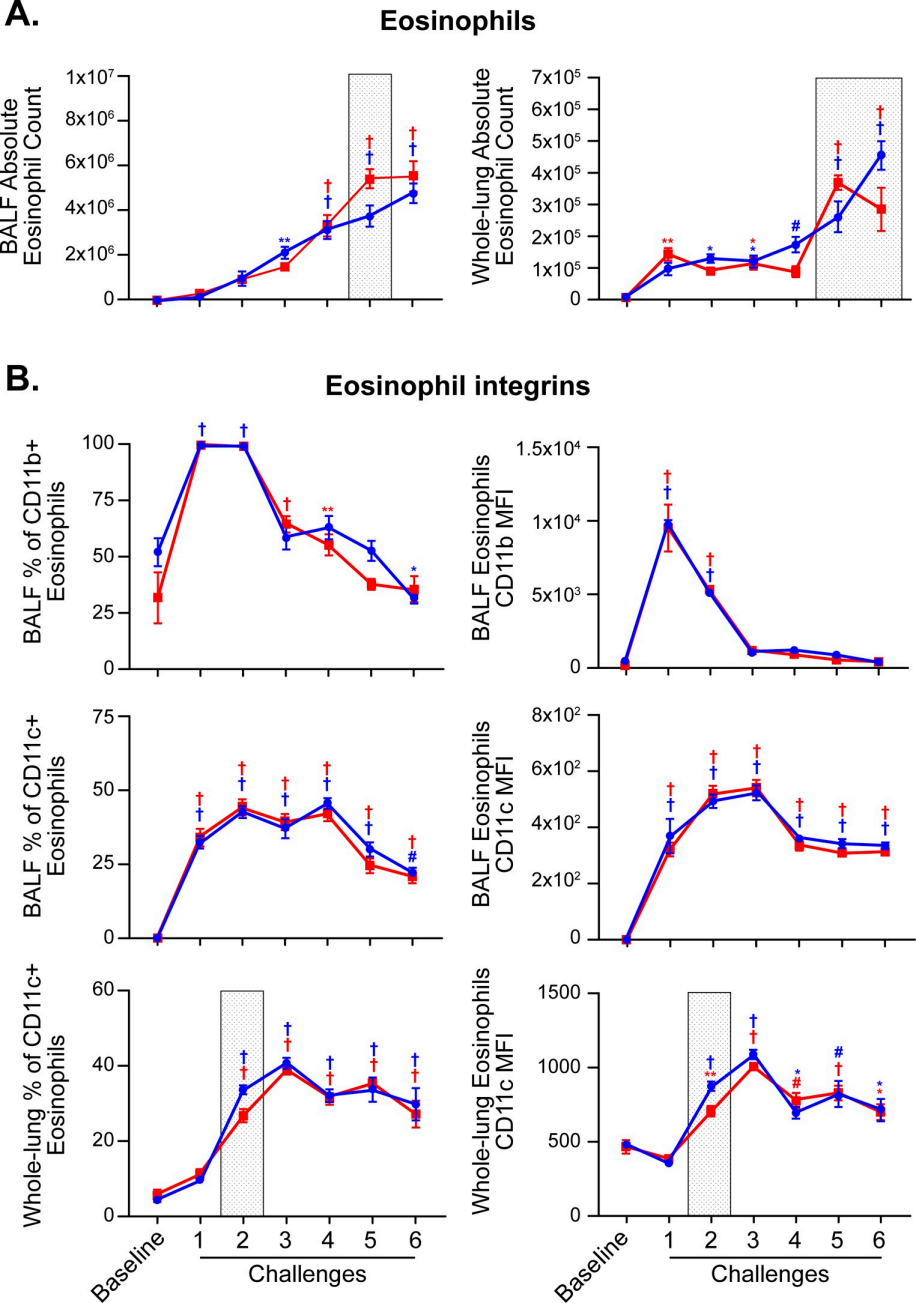
Sexual dimorphism in BALF and whole-lung eosinophil endpoints during allergic inflammation. (A) Quantification of eosinophils in bronchoalveolar lavage and lung homogenates of male and female mice by multi-color flow cytometry. (B) Assessment of eosinophil integrins CD11b and CD11c by multi-color flow cytometry (% and MFI data). Symbols in blue represent males and symbols in red represent females. When comparing challenge time point responses (1–6) relative to baseline (for either male or female data) by two-way ANOVA: *p < 0.05; **p < 0.01; #p < 0.001; †p < 0.0001. Gray shaded boxes represent a statistically significant difference (p < 0.05) between males and females at that particular time-point. N = 4 mice of each sex per time point for “Baseline” and “Challenge 6” groups, N = 8 mice/sex per time point for “Challenge 1–5” groups/time points. Data are from a single experiment representative of two independent experiments.

**Fig 3:**
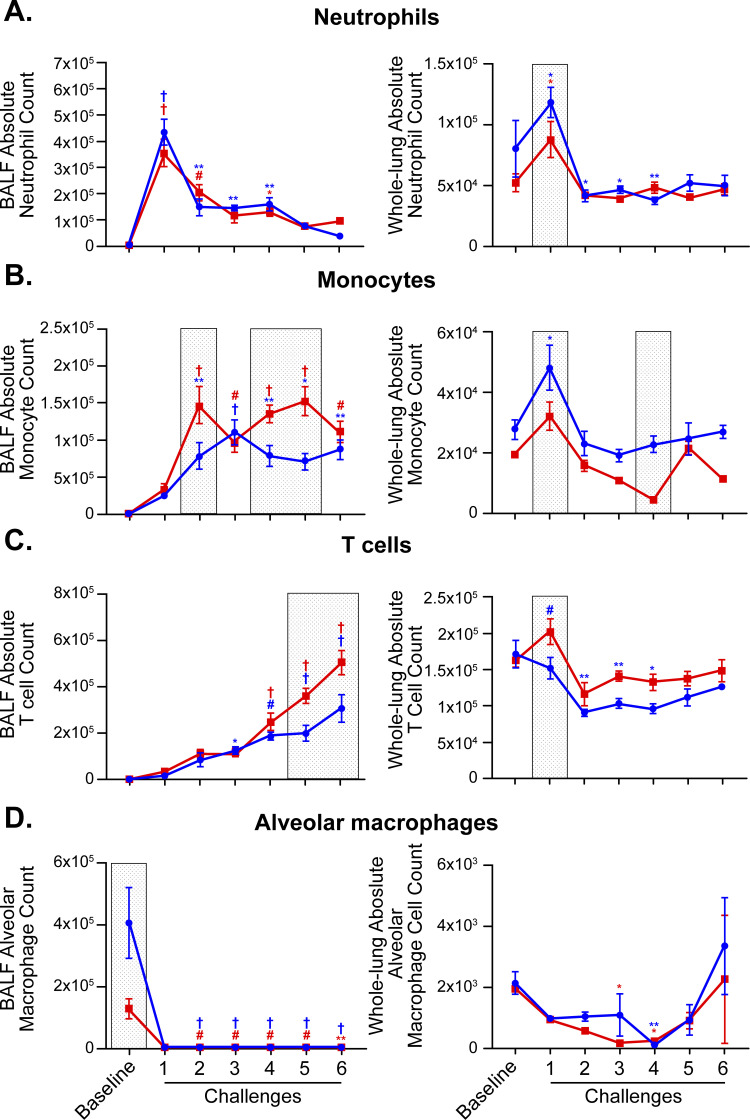
Sex differences in BALF and lung tissue neutrophils, monocytes, T cells, and alveolar macrophages during allergic inflammation. Quantification of neutrophils (A), monocytes (B), T cells (C), and alveolar macrophages (D) in bronchoalveolar lavages (left) and lung homogenates (right) of male and female mice by multi-color flow cytometry. Data represented as absolute cell counts. When comparing challenge time point responses (1–6) relative to baseline (for either male or female data) by two-way ANOVA: *p < 0.05; **p < 0.01; #p < 0.001; †p < 0.0001. Gray shaded boxes represent a statistically significant difference (p < 0.05) between males and females at that particular time-point. N = 4 mice of each sex per time point for “Baseline” and “Challenge 6” groups, N = 8 mice/sex per time point for “Challenge 1–5” groups. Data are from a single experiment representative of two independent experiments.

Changes in eosinophil numbers during allergic inflammation clearly illustrate how sexual dimorphism can be both time and tissue compartment dependent (**Fig 2A**). Lung absolute eosinophil counts showed sex differences that were also time dependent. In challenge 5, female mice had higher numbers of absolute eosinophil counts when compared to male mice. The opposite was true in challenge 6. When analyzing the BALF compartment, female mice had a significantly higher percentage of eosinophils in challenge 1 when compared to male mice (**S1 Fig**). The results of the subsequent challenge showed the opposite pattern: male mice had a significantly higher percentage of eosinophils. When looking at the lung tissue compartment, male mice had a higher percentage of eosinophils in challenges 4 and 6 when compared to female mice. Homing of eosinophils to different compartments and their trans-endothelial and trans-epithelial recruitment depends on expression of specific integrins. Thus, we profiled two integrins: CD11b and CD11c, which are typically implicated in submucosal (CD11b) and mucosal (CD11c) adhesion and activation of tissue eosinophils (**Fig 2B**). There was no difference between sexes in BALF eosinophils that were CD11b+ or CD11c+. In challenge 2, male mice had a higher percentage of whole-lung eosinophils that were CD11c+ and a higher eosinophil CD11c MFI when compared to female mice. Early in the kinetic study (challenge 1), male mice had an increased percentage of neutrophils both in the BALF and whole-lung compartments when compared to female mice (**Fig 3A**). Monocyte populations also demonstrated significant compartment-specific sexual dimorphism (**Fig 3B**). In the BALF compartment, female mice had higher percentage of monocytes (challenge 2) and absolute monocytes counts (challenges 2, 4, and 5) when compared to male mice. In contrast, in the whole-lung compartment, male mice showed higher percentages of monocytes (challenge 1, 3, and 4) and absolute monocyte counts (challenge 1 and 4) when compared to female mice. In both compartments, T cell numbers were higher in female mice at multiple time points of the kinetic study (**Fig 3C**). Alveolar macrophage counts were not significantly different between sexes during the course of inflammation, although males had significantly higher numbers of alveolar macrophages in BALF at baseline (**Fig 3D**).

### Sexual dimorphism in lung cytokine, chemokine and tissue marker expression

**Fig 4:**
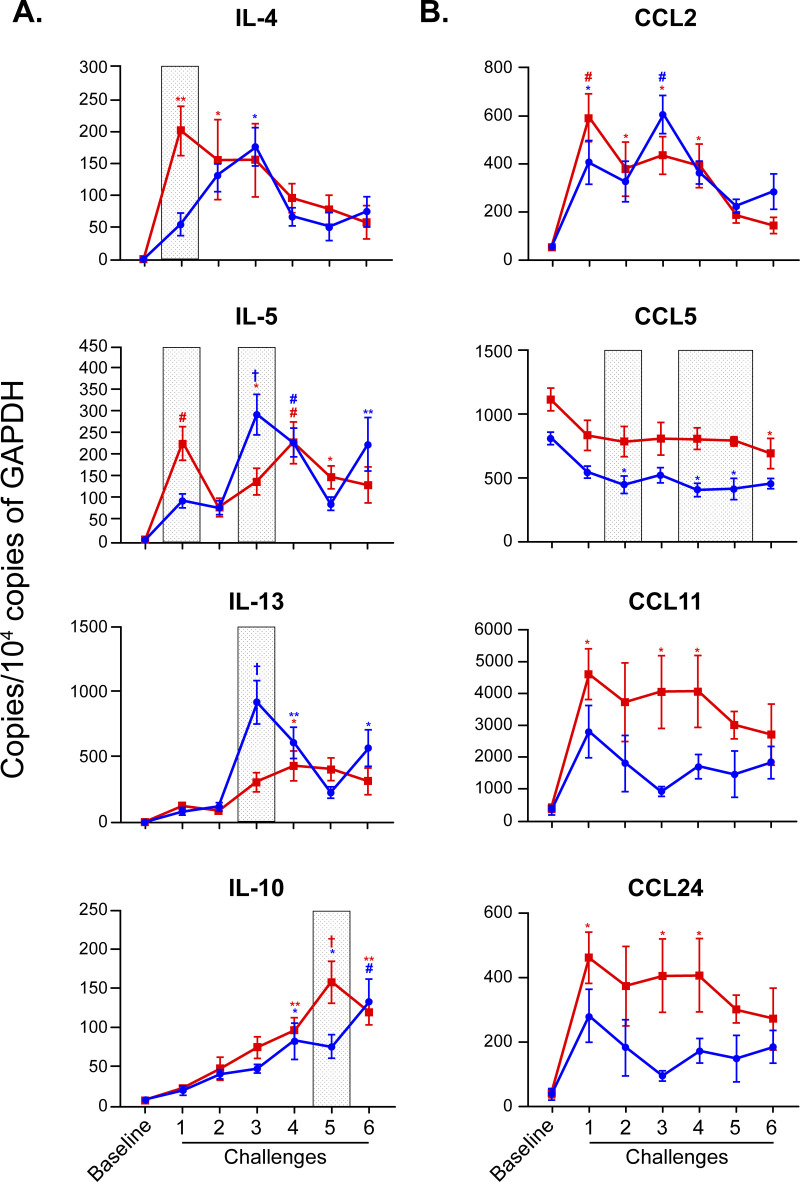
Sexual dimorphism in lung tissue expression of cytokines and chemokines in an OVA model. (A) Expression of Type 2 cytokines IL-4, IL-5, IL-13, and IL-10 by qPCR in lung tissue. (B) Expression of chemokines Ccl2, Ccl5, Ccl11, and Ccl24 by qPCR in lung tissue. The housekeeping gene was glyceraldehyde-3-phosphate dehydrogenase (GAPDH). Two-way ANOVA: *p < 0.05; **p < 0.01; #p < 0.001; †p < 0.0001 for assessment of time responses (challenge vs. baseline). Gray shaded boxes represent statistically significant sexual dimorphism (p < 0.05) for any given time point in the model. N = 4 mice of each sex per time point for “Baseline” and “Challenge 6” groups, N = 7 mice/sex per time point for “Challenge 1–5” groups. Data are from a single experiment representative of two independent experiments.

**Fig 5:**
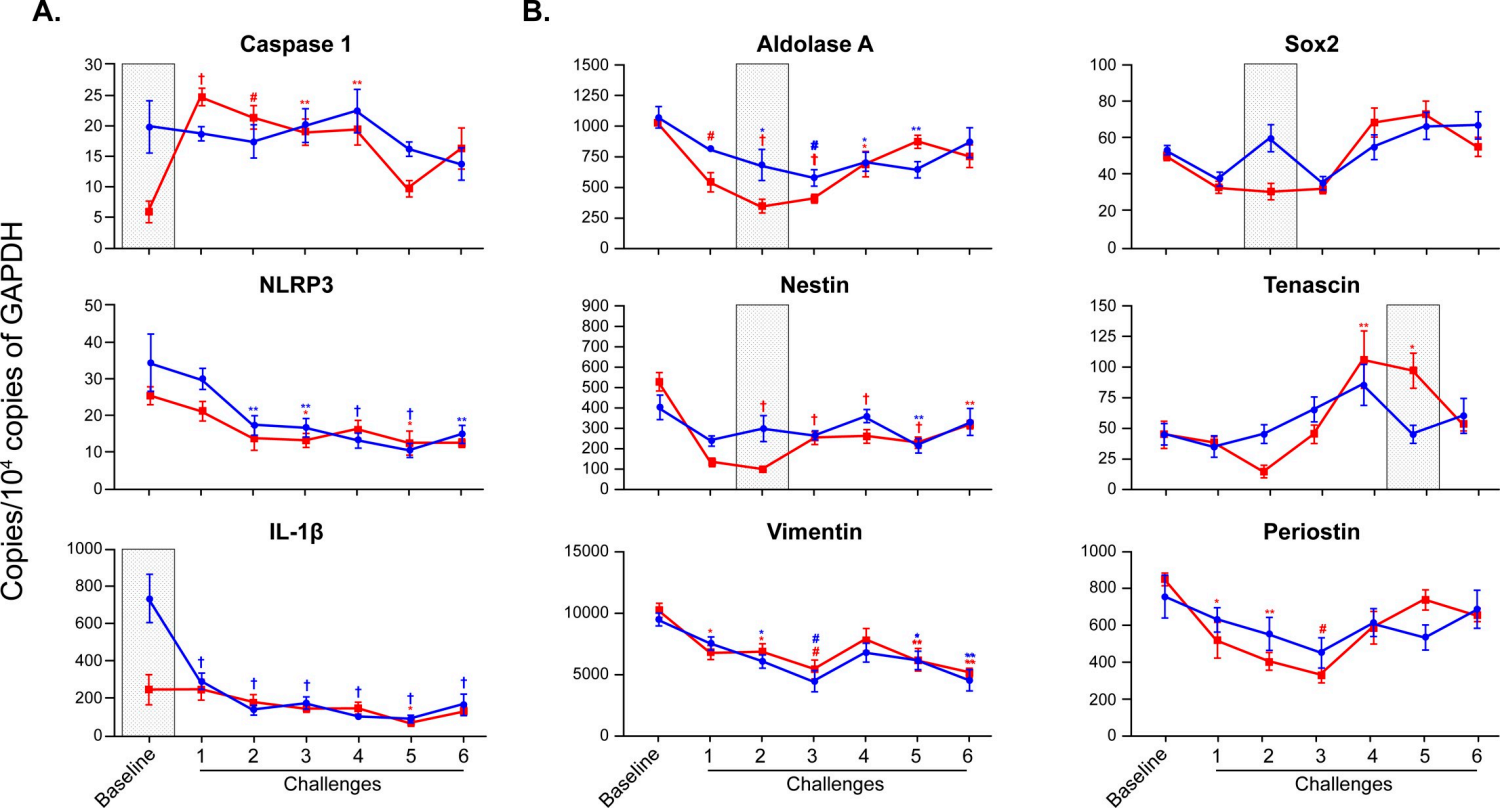
Sex differences in lung tissue expression of inflammasome and tissue injury and repair markers during allergic inflammation. (A) mRNA expression of inflammasome markers Caspase-1, NLRP3, and IL-1β in lung tissue. (B) Expression of tissue injury/repair markers Aldolase A, Sox2, Nestin, Tenascin-C, Vimentin, and Periostin in lung tissue. Two-way ANOVA: *p < 0.05; **p < 0.01; #p < 0.001; †p < 0.0001 for assessment of time responses (challenge vs. baseline). Gray shaded boxes represent statistically significant sexual dimorphism (p < 0.05) for any given time point in the model. N = 4 mice of each sex per time point for “Baseline” and “Challenge 6” groups, N = 7 mice/sex per time point for “Challenge 1–5” groups. Data are from a single experiment representative of two independent experiments.

Lung expression of cytokines (**Fig 4A**) and chemokines (**Fig 4B**) also showed sex differences. These sex differences changed throughout the kinetic study. In challenge 1, female mice had higher levels of IL-5 expression when compared to male mice. In challenge 3, the opposite was true. In addition, female mice had significantly higher levels of whole-lung IL-4 in challenge 1, while male mice had higher levels of whole-lung IL-13 in challenge 3. Furthermore, female mice had significantly higher levels of whole-lung IL-10 in challenge 5 and higher levels of whole-lung Ccl5 in challenges 2, 4, and 5 when compared to their male littermates. Although not statistically significant, there is a trend showing higher levels of Ccl24 (**Fig 4B**) and IL-33 (**S2 Fig**) in female mice when compared to males. Both Ccl5 and Ccl24 are key chemokines for the recruitment of eosinophils to the mucosal compartment. There was no statistically significant sexual dimorphism in Ccl2 and Ccl11, which are important for the trans-endothelial migration of several leukocytes. Male mice have significantly higher baseline levels of inflammasome markers caspase 1 and IL-1β when compared to females (**Fig 5A**). Finally, female mice had lower levels of markers Fgfr1 and Fgfr2 (growth factors regulatory for epithelium) (**S3 Fig**), Aldolase A (glycolytic marker), Sox2 (epithelial differentiation inducer), and Nestin (mesenchymal marker) in challenge 2 when compared to males (**Fig 5B**). Tenascin C (Tnc), an extracellular matrix protein associated with eosinophil accumulation in tissue, showed sex dimorphism later in the kinetic study, with higher levels seen in female mice (challenge 5) (**Fig 5B**).

### B cells and humoral response

**Fig 6:**
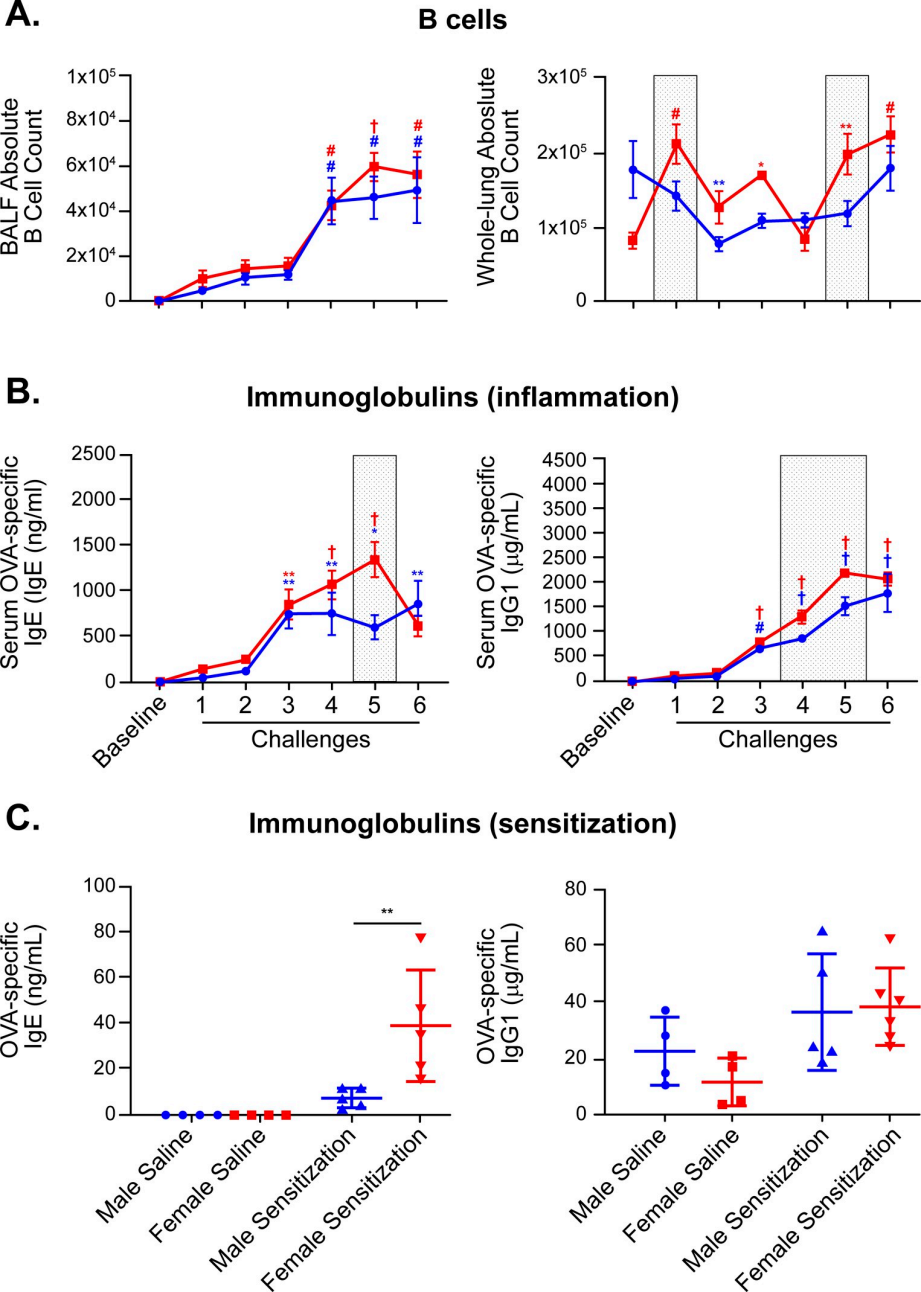
Sexual dimorphism in B cells, OVA-specific IgE, and serum OVA-specific IgG1 immunoglobulins. (A) Quantification of B cells in bronchoalveolar lavage and lung tissue of male and female mice by multi-color flow cytometry. (B) Measurements of serum OVA-specific IgE and OVA-specific IgG1 during challenge phase of the model (inflammation). (C) Measurements of serum OVA-specific IgE and IgG1 following intraperitoneal antigen sensitization and prior to the first OVA challenge (sensitization). Two-way ANOVA: *p < 0.05; **p < 0.01; #p < 0.001; †p < 0.0001 for assessment of time responses (challenge vs. baseline). Gray shaded boxes represent statistically significant sexual dimorphism (p < 0.05) for any given time point in the model. For graphs in **A.** and **B.**: N = 4 mice of each sex per time point for “Baseline” and “Challenge 6” groups, N = 6 mice/sex per time point for “Challenge 1–2” groups, N = 8 mice/sex per time point for “Challenge 3–5” groups. Data are from a single experiment representative of at least two independent experiments. For graphs in **C.**: N = 4 mice/sex for “Saline” groups, N = 5 mice/sex for “Sensitization” groups. Data are from a single experiment.

In lung tissue, female mice had higher percentages of B cells in challenges 2, 3, 5, and 6. Females also showed higher absolute B cell numbers in challenges 1 and 5 when compared to male mice (**Fig 6A**). Even though it did not reach statistical significance, there was also a trend towards higher numbers of B cells in the BALF compartment of female mice. The sex dimorphism in B cell number was consistent with the immunoglobulin response differences measured in male and female mice. During challenge-induced inflammatory response, female mice had higher levels of OVA-specific IgE in challenge 5 and OVA-specific IgG1 in challenges 4 and 5 when compared to male mice (**Fig 6B**). Moreover, OVA-specific IgE was significantly elevated in females compared to males one week after sensitization, but prior to the first antigen challenge, demonstrating sexual dimorphism in the processes of antigen sensitization (**Fig 6C**). Post-sensitization, there was no sex difference in OVA-specific IgG1 levels (**Fig 6C**).

## Discussion

This study is the most complete time course assessment to date of the sex differences underlying the lung allergic inflammatory responses in a standard murine OVA model of asthma. A significant strength of this model is that it elicits robust T- and B-cell driven type 2 responses. Furthermore, it replicates several features of human asthma, including eosinophilic airway inflammation, goblet cell hyperplasia, increased airway hyperresponsiveness, and elevated allergen-specific IgE levels. Nevertheless, it is important to note that this model has known limitations. These include an intraperitoneal route for allergen sensitization and the need for an aluminum-containing adjuvant. Furthermore, unlike the house dust mite or *Alternaria alternata*-induced mouse models of asthma, inflammation is not driven by a clinically-relevant antigen. Additionally, group 2 innate lymphoid cells are dispensable in this model [22] and it is possible to induce some degree of tolerance [8]. Despite these limitations, the murine OVA model of asthma remains a primary choice for studying the processes of allergic inflammation, leukocyte recruitment and adaptive immune responses in asthma. It is also a popular choice for modeling sex differences in allergic inflammation [10–19]. Our study is novel in its choice to investigate sex differences kinetically and in different tissue compartments, thus elucidating spatio-temporal development of sex differences during inflammation. Moreover, we paid extra attention to baseline differences between sexes, and tissue repair and metabolism markers in addition to the traditional scoring of inflammatory parameters.

It is almost a convention now to report that females have higher Type 2 responses in allergic models than male mice [23]. Table 1 illustrates how the results of our study match the endpoints of studies using BALB/cJ mice (strain that we used), which all report higher numbers of eosinophils and Type 2 cytokine responses in females. Surprisingly, however, no published studies of BALB/cJ mice employed the conventional 3-challenge model but rather used 1, 5, 8, 10, or 15 ovalbumin challenges. If 2–3 challenges were done in these studies, the conclusions would have been the opposite, as we observed significantly higher Type 2 readouts (% of BALF eosinophils, lung IL-5 and IL-13 mRNA levels) in male rather than female mice in this time frame. Sex differences in all measured parameters at all time points in the asthma model are summarized in **Table 2**. Overall, it is apparent that male biased dimorphism is more predominant in early antigen challenge responses and innate parameters, while female biased dimorphism is more evident in adaptive immune parameters and late phase tissue repair and resolution responses. Therefore, reports of sexual dimorphism can be somewhat subjective based on the time points and sampling sites chosen in the study. If only one specific time point in a model is chosen for measuring inflammatory end points, the statistical significance of observed changes is also going to be higher. In this study, we used more complex statistical tests accounting for repeated measures over the time course of the model and multiple comparison adjustments, which only preserved the most significant sex differences in the duration of allergic inflammatory response. We hope that investigators can use data in this report as a guide to choosing treatment timelines in their ovalbumin models of asthma.

**Table 2:** Schematic summary of statistically significant sex differences in all measured endpoints of this study. Blue denotes male-biased dimorphism when responses were greater in male mice compared to female mice. Red indicates female-biased dimorphism. Empty cells indicate no statistically significant differences between males and females. BALF: bronchoalveolar lavage fluid; WL: whole lung.

	Baseline	Challenge 1	Challenge 2	Challenge 3	Challenge 4	Challenge 5	Challenge 6
BALF Eosinophils (% of CD45+)							
BALF Absolute Eosinophil Count							
BALF Neutrophils (% of CD45+)							
BALF Monocytes (% of CD45+)							
BALF Absolute Monocyte Count							
BALF T Cells (% of CD45+)							
BALF Absolute T Cell Count							
WL Eosinophils (% of CD45+)							
WL Absolute Eosinophil Count							
WL % CD11c+ Eosinophils							
WL Eosinophil CD11c MFI							
WL Neutrophils (% of CD45+)							
WL Absolute Neutrophil Count							
WL Monocytes (% of CD45+)							
WL Absolute Monocyte Count							
WL T Cells (% of CD45+)							
WL Absolute T Cell Count							
WL B Cells (% of CD45+)							
WL Absolute B Cell Count							
WL IL-4 mRNA							
WL IL-5 mRNA							
WL IL-13 mRNA							
WL IL-10 mRNA							
WL CCL5 mRNA							
WL Caspase-1 mRNA							
WL IL-1β mRNA							
WL FGFR1 mRNA							
WL FGFR2 mRNA							
WL Aldolase A mRNA							
WL Sox2 mRNA							
WL Nestin mRNA							
WL Tenascin mRNA							
Serum OVA-specific IgE							
Serum OVA-specific IgG							

From the statistically robust kinetic patterns measured in our study, several important insights can be gained into the biology of sex differences unfolding during an inflammatory response. At baseline, we found that females have a proportionally higher representation of T cells but lower percentage of B cells compared to males in normal resting lung tissue. Interestingly, lung mRNA levels of inflammasome genes *IL-1β*, *Cas1* and *Nlrp3* was higher in males at baseline. A previous study identified sexual dimorphism in postoperative pain regulation by NLRP3 inflammasome [24]. It has also been published that estrogen can downregulate CD16 expression and downstream IL-1β [25]. Interestingly, in an OVA model of allergic inflammation, 17β-estradiol treatment suppressed airway inflammation via negative regulation of NLRP3 inflammasome activation [26]. However, we found higher levels of OVA-specific IgE in females immediately after intraperitoneal antigen sensitization. This suggests that sex differences manifest themselves early and may introduce sex bias in T cell polarization and skewing of adaptive immune responses. What sex differences in inflammasome activity and sensitization could mean for the inception of allergic responses needs further investigation.

There were multiple sex differences in inflammatory response during the antigen challenge phase of the model. In summary, males had more dominant innate immune responses (recruitment of neutrophils and monocytes, lung mRNA levels of inflammasome genes), while females exhibited higher adaptive immune responses, evidenced by increased OVA-specific IgE, T and B cell recruitment, and lung IL-4 mRNA throughout the model. Estradiol has been shown to inhibit MCP-1-induced monocyte migration [27] and fMLP-induced neutrophil migration [28] via estrogen receptor alpha (ERα), which may explain the lesser recruitment of these cells in females in our model. Noteworthy, sexual dimorphism in monocyte recruitment was strikingly compartment-dependent, where males had higher monocyte counts in lung tissue, but females had more monocytes in BALFs, which may implicate the differential action of sex hormones in different tissue compartments.

Contrariwise, the most consistent female-biased sexual dimorphism was found in the B and T cell populations and in serum levels of OVA-specific IgE and IgG1. This is consistent with studies reporting that estrogen promotes the survival and activation of B cells [29], interferes with B cell tolerance [30], and enhances immunoglobulin production [31–34]. Female humans also have higher numbers of several B cell subsets in PBMCs [35]. In mice sensitized with phospholipase A2 (PLA2), females have higher levels of PLA2-specific IgE than male mice. Castrated male mice had higher levels of PLA2-specific IgE than sham-operated mice and testosterone reduced the levels of PLA2-specific IgE in these castrated mice [36]. In part this may be due to the regulatory effect of estrogen on B cell isotype switching via an IL-4-dependent mechanism, as estrogen increases the expression of IL-4 and GATA-3 [37, 38]. In support of these reports, compared to males, we found significantly increased IL-4 mRNA in the lung tissue of females after the first antigen challenge as well as a sustained increase in serum OVA-specific IgE throughout the entire model. On the contrary, androgens suppress IL-4 and B cell development [39–41] and can inhibit immunoglobulin production [42]. Castrated males have higher levels of IL-4 from splenic cells when compared to sham-operated males [12]. Finally, ERα is equally expressed in CD4+ T cells [43] and there is evidence that estrogen may promote T-cell priming against specific antigens [17] and the expansion of antigen-specific CD4+ T cells [44].

The “classical” ovalbumin challenge model we used in this study is largely acute in nature and is self-resolving even in presence of continuous antigen challenges due to the development of tolerance [45–47]. The late phase of our model (challenges 4–6) represents lung repair and resolution of inflammation responses. This phase is characterized by peak recruitment of eosinophils in both compartments, T and B cells in BALFs, as well as peak lung mRNA levels of eosinophil-attracting chemokines Ccl5, Ccl11 and Ccl24, cytokines IL-10 and IL-33, and epithelial repair markers such as *Igf1*, *Sox2* and *Tnc*. We found that females had significantly higher responses in this phase of the model, including eosinophils and expression of eosinophil-attracting chemokines and IL-10. Estrogen has been previously shown to have eosinophil-promoting activity [48]. Estrogen receptor agonists caused increased infiltration of eosinophils in a murine model of allergic airway inflammation [49]. Estrogen promotes eosinophilic infiltration into the rat uterus during the estrous cycle [50, 51]. Male mice treated with estradiol had higher levels of blood and lung eosinophilia in a murine model of allergic asthma [43] and castrated males had higher levels of BALF eosinophils when compared to sham-operated males [12]. In contrast, testosterone reduced eosinophil adhesion to human mucosal microvascular endothelial cells and eosinophil viability [52]. Estrogen has also been shown to promote the secretion of IL-10 and induction of regulatory B cells [53] and the production of CCL2 and CCL5 [54, 55], as well as regulate the expression and function of chemokine receptors [56]. Conventionally, elevated eosinophil counts are thought to indicate worsened allergic inflammation. However, in acute model settings, this may indicate a beneficial response of enhanced inflammation resolution. This idea is supported by a report by Takeda et al. [57] that demonstrated that, in this exact mouse ovalbumin model, IL-10 derived from eosinophils was necessary for the resolution of allergic inflammation. Given these observations, an interesting possibility is that increased inflammation and disease prevalence in females may reflect a greater wound healing response. Although beneficial in acute mouse model settings, the perpetuated injury and healing response in chronic asthma would result in unwanted loss of epithelial barrier function and tissue remodeling. Thus, it would be relevant for future studies to examine sex differences in wound healing and tissue remodeling in health and disease, and validate markers of such processes at the protein level.

Finally, it is important to note that sex hormones could play a direct role in modulation of various immune pathways and mediators in this asthma model, mechanisms of which requires further investigation. For instance, estrogen can promote the migration of eosinophils to the airways during allergic inflammation [43, 49]. In contrast, androgen reduces eosinophil viability and their adhesion to endothelial cells [52]. Estrogen can also stimulate the expansion of antigen-specific CD4+ T cells and increase the secretion of IL-4 and expression of GATA3 in these cells [38, 44]. Furthermore, estrogen can enhance B cell survival and immunoglobulin production [29, 31], while androgen suppresses B cell maturation and IgE class switching [12, 41]. Finally, estrogen has been also shown to stimulate the migration of monocytes [58]. Thus, the effects of sex hormones can in part explain the sex differences in immune cells observed in our murine model of asthma.

In summary, sexual dimorphism in airway inflammation is both time and tissue compartment dependent. Varying sex difference patterns in different stages of the model may be reflective of the different roles the estrogen and androgen pathways play in sensitization, innate vs. adaptive immune responses, and resolution of inflammation. An important take away from this study is that the spatiotemporal variability in sex differences in a murine model of asthma must be accounted for when planning experiments to model sex bias in allergic inflammation.

## Supporting information

S1 Fig Sex differences in BALF and lung tissue neutrophils, monocytes, T cells, and alveolar macrophages during allergic inflammation. Quantification of neutrophils (A), monocytes (B), T cells (C), and alveolar macrophages (D) in bronchoalveolar lavages (left) and lung homogenates (right) of male and female mice by multi-color flow cytometry. Data represented as percentages of all hematopoeitcic CD45(+) cells. When comparing challenge time point responses (1–6) relative to baseline (for either male or female data) by two-way ANOVA: *p < 0.05; **p < 0.01; #p < 0.001; †p < 0.0001. Gray shaded boxes represent a statistically significant difference (p < 0.05) between males and females at that particular time-point. N = 4 mice/sex per group for “Baseline” and “Challenge 6” groups, N = 8 mice/sex per time point for “Challenge 1–5” groups. Data are from a single experiment representative of two independent experiments.
(TIF)

S2 Fig No sexual dimorphism in lung tissue expression of IL-33 during challenge phase of OVA model by qPCR. Two-way ANOVA: *p < 0.05; **p < 0.01; #p < 0.001; †p < 0.0001 for assessment of time responses (challenge vs. baseline). N = 4 mice of each sex per time point for “Baseline” and “Challenge 6” groups, N = 7 mice/sex per time point for “Challenge 1–5” groups. Data are from a single experiment representative of two independent experiments.
(TIF)

S3 Fig Sex differences in lung tissue expression of growth factors during allergic inflammation by qPCR. Two-way ANOVA: *p < 0.05; **p < 0.01; #p < 0.001; †p < 0.0001 for assessment of time responses (challenge vs. baseline). Gray shaded boxes represent statistically significant sexual dimorphism (p < 0.05) for any given time point in the model. N = 4 mice of each sex per group for “Baseline” and “Challenge 6” groups, N = 7 mice/sex per time point for “Challenge 1–5” groups. Data are from a single experiment representative of two independent experiments.
(TIF)
