# Supplementary figures and images for: Sex differences in a murine model of asthma are time and tissue compartment dependent
Source: PLoS One. 2023 Oct 11;18(10):e0271281. doi: 10.1371/journal.pone.0271281 (PMC10566727; doi:10.1371/journal.pone.0271281)

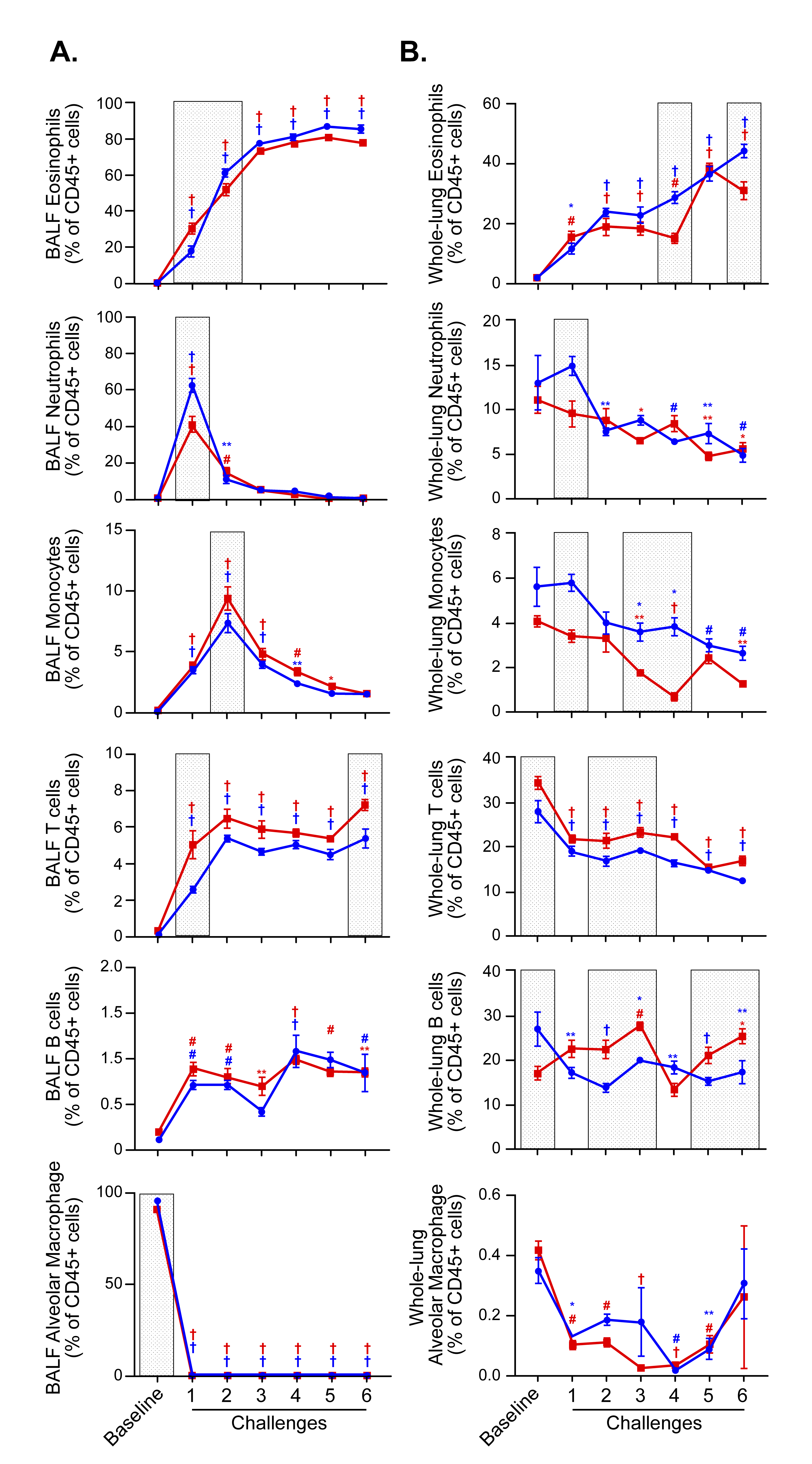

Supplement: S1 Fig — Quantification of neutrophils (A), monocytes (B), T cells (C), and alveolar macrophages (D) in bronchoalveolar lavages (left) and lung homogenates (right) of male and female mice by multi-color flow cytometry. Data represented as percentages of all hematopoeitcic CD45(+) cells. When comparing challenge time point responses (1–6) relative to baseline (for either male or female data) by two-way ANOVA: *p < 0.05; **p < 0.01; #p < 0.001; †p < 0.0001. Gray shaded boxes represent a statistically significant difference (p < 0.05) between males and females at that particular time-point. N = 4 mice/sex per group for “Baseline” and “Challenge 6” groups, N = 8 mice/sex per time point for “Challenge 1–5” groups. Data are from a single experiment representative of two independent experiments. (TIF) [file pone.0271281.s001.tif]

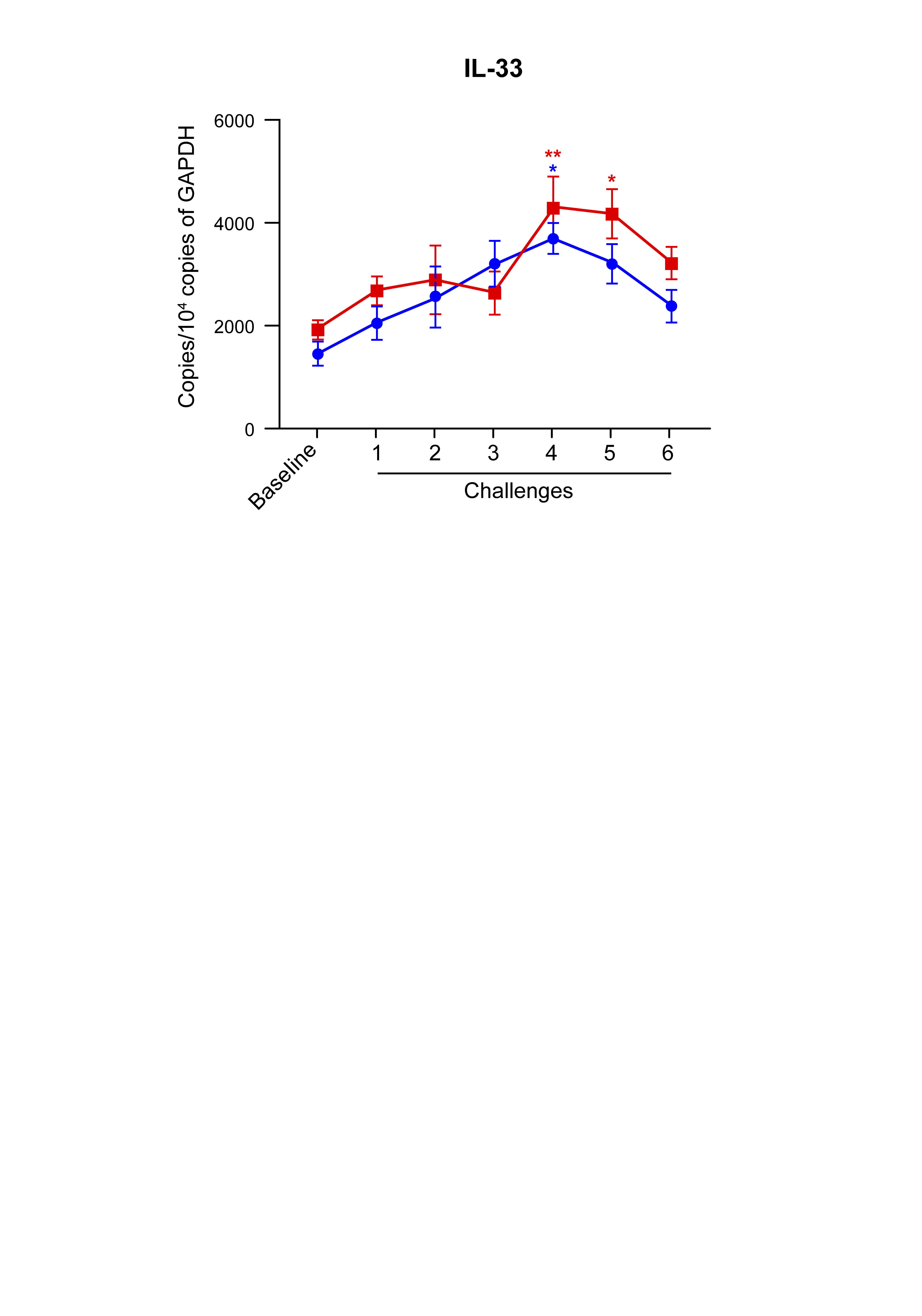

Supplement: S2 Fig — Two-way ANOVA: *p < 0.05; **p < 0.01; #p < 0.001; †p < 0.0001 for assessment of time responses (challenge vs. baseline). N = 4 mice of each sex per time point for “Baseline” and “Challenge 6” groups, N = 7 mice/sex per time point for “Challenge 1–5” groups. Data are from a single experiment representative of two independent experiments. (TIF) [file pone.0271281.s002.tif]

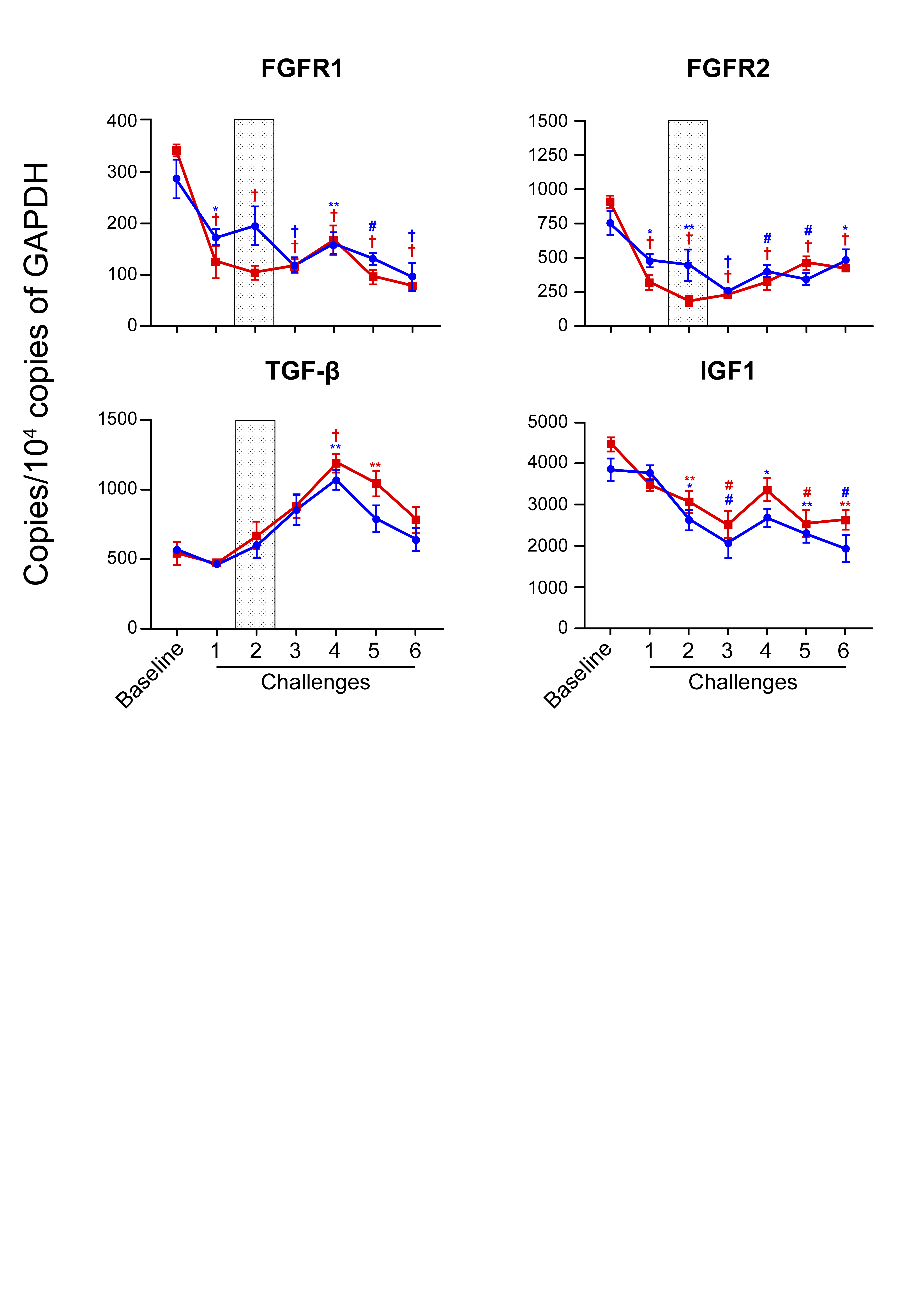

Supplement: S3 Fig — Two-way ANOVA: *p < 0.05; **p < 0.01; #p < 0.001; †p < 0.0001 for assessment of time responses (challenge vs. baseline). Gray shaded boxes represent statistically significant sexual dimorphism (p < 0.05) for any given time point in the model. N = 4 mice of each sex per group for “Baseline” and “Challenge 6” groups, N = 7 mice/sex per time point for “Challenge 1–5” groups. Data are from a single experiment representative of two independent experiments. (TIF) [file pone.0271281.s003.tif]
